# The effectiveness of oral bovine lactoferrin compared to iron supplementation in patients with a low hemoglobin profile: A systematic review and meta-analysis of randomized clinical trials

**DOI:** 10.1186/s40795-023-00818-6

**Published:** 2024-01-30

**Authors:** Maria-Dolores Christofi, Konstantinos Giannakou, Meropi Mpouzika, Anastasios Merkouris, Maria Vergoulidou – Stylianide, Andreas Charalambous

**Affiliations:** 1 Department of Nursing, Faculty of Health Sciences, Cyprus University of Technology, 15 Vragadinou, 3041 Limassol, Cyprus; 2 Department of Health Sciences, School of Sciences, European University Cyprus, 6 Diogenous, 2404 Nicosia, Cyprus; 3 Hematology-Oncology Center, 33, Ayias Zonis, 3027 Limassol, Cyprus

**Keywords:** Lactoferrin, Hemoglobin, Iron deficiency anemia, Bovine lactoferrin, Iron, Ferrous sulfate, Systematic review

## Abstract

**Background:**

Patients with a low serum blood hemoglobin concentration suffer from a pathologic state that contributes significantly to morbidity and mortality figures worldwide. Oral iron supplementation, the most common method of treatment, is reported to have poor patient adherence, due to its unwanted side effects. Lactoferrin is a globular glycoprotein of the transferrin family that has shown promising results in patients with a low hemoglobin profile. This systematic review and meta-analysis of randomized clinical trials explore its effect on blood hemoglobin compared to conventional iron preparations.

**Methods:**

We followed the PRISMA Guidelines for reporting systematic reviews and meta-analyses. A systematic search was conducted in electronic databases (PubMed, CINAHL, Scopus, and Cochrane) from inception to June 2022. Meta-analysis was performed on studies where the primary outcome was the mean Hb concentration, comparing lactoferrin to ferrous sulfate subgroups. We assessed the methodological quality of the trials using the Jadad scoring scale.

**Results:**

Nineteen trials published between 2006 and 2022 met the eligibility criteria. It has been found that the levels of Hb concentration in different populations with varying health conditions undergo a moderate to significant change after treatment with all types of trialed interventions, including both iron and lactoferrin treatment, in both the intervention group and the comparison group. Most of the studies report that LF showed a statistically significant increase in Hb concentration levels, compared to those in the iron group. The meta-analysis included seven trials comparing the effectiveness of lactoferrin to ferrous sulfate for patients with low Hb concentration. The analysis showed a statistically significant increase in Hb levels in the oral bovine lactoferrin group compared to ferrous sulfate (SMD -0.81, 95% CI: -1.21, -0.42, *p* < 0.0001, I^2^ = 95.8%, P heterogeneity < 0.001).

**Conclusions:**

Lactoferrin is an effective intervention at doses of 100–250 ng/day, for patients with a low Hb concentration. As a safer option and with high compliance evidence, lactoferrin can serve as an iron replacement treatment for patients who may be experiencing adverse side effects due to iron intake.

## Background

When the number of red blood cells is insufficient to support the body’s metabolic needs, it leads to anemia, a pathologic state defined by a low hemoglobin concentration, which contributes significantly to morbidity and mortality figures worldwide [1, 2]. In 2019, the estimated global prevalence of anemia in women and children, based on the distribution of blood hemoglobin concentration values in different populations globally, was strikingly reported as 39.8% in children up to five years of age, 29.9% in women of reproductive age (non-pregnant), and 36.5% in pregnant women [3]. Oral iron supplementation is the most common conventional method of treatment. However, it usually causes unwanted gastrointestinal side effects in patients in 70% of cases, which may consequently affect the patients’ adherence to treatment [4, 5].

Lactoferrin (LF) is a globular glycoprotein of the transferrin family, possessing a high iron binding affinity, and it is structurally and chemically similar to serum transferrin [6]. Bovine LF (bLF) shares a high degree of similarity (up to 70%), with human lactoferrin, which is found in body exocrine secretions (human milk, tears, and saliva) and in the secondary granules of neutrophils [7, 8]. It has shown promising results in many studies evaluating its effect on blood hemoglobin levels in women, children, and patients with chronic disease [9].

A comprehensive analysis of the randomized trials conducted to date, examining the effects of oral bLF as a supplement on hemoglobin, in different populations and health states, is needed to evaluate and clarify the effect and safety of oral LF supplementation. Therefore, the scope of this systematic review and meta-analysis is to describe its effect on blood hemoglobin and to quantify the effect of oral bLF on hemoglobin in different populations and health states for both genders.

## Methods

### Information sources and search strategy

For conducting this systematic review and meta-analysis, the PRISMA Guidelines for reporting systematic reviews and meta-analyses (PRISMA) were followed [10]. The protocol of this study was registered with the International Prospective Register of Systematic Reviews (PROSPERO) (CRD42022348383).

**Fig. 1:**
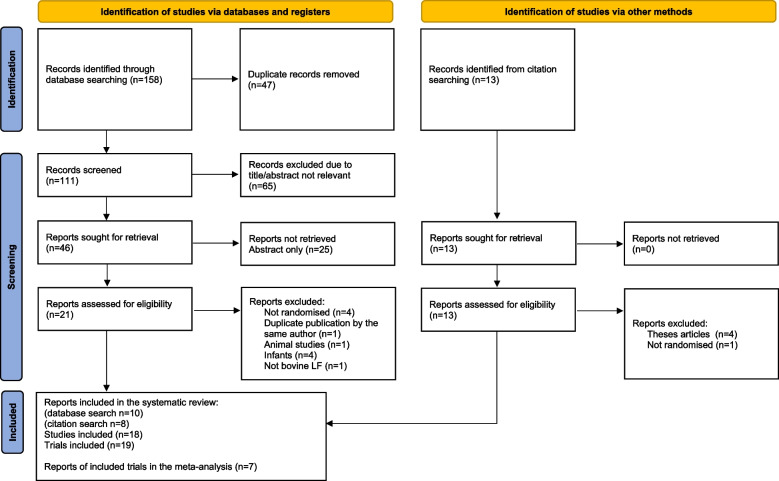
PRISMA flow diagram

The following electronic databases were independently searched by two researchers (MC, AC) from inception to June 2022: PubMed, CINAHL, Scopus, Cochrane and Embase. Studies were retrieved by using the following ‘Medical Subject Heading’ (MesH) and text words combined using Boolean operators as a search strategy for each electronic database: (Lactoferrin OR ‘‘bovine lactoferrin’’ OR lactotransferrin) AND (‘‘anemia treatment’’ OR ‘‘anaemia trea tment’’ OR ‘‘iron deficiency anemia’’ OR ‘‘iron deficiency anaemia’’ OR anaemia OR anemia OR anemic OR anaemic OR ‘‘iron deficiency’’ OR hemoglobin OR haemoglobin). The search was limited to title and abstract and clinical trial articles, and articles published in English. Following the completion of the search, the title and abstract of all the retrieved articles were screened for their relevancy to the subject being studied, and articles were eliminated if irrelevant. Finally, the full text of the potentially eligible articles was examined for potential inclusion in the analysis. Disagreements were settled through mediation and discussion with a third author (KG). The citation lists of the articles retrieved were manually checked to identify similar studies. The database search strategy is presented in Fig. 1.

### Eligibility criteria

We used the Participants, Intervention, Comparison, Outcomes, and Study design (PICOS) approach to identify included studies [11]. Eligible studies included male and female adult participants and children ≥ 2 years of age. The clinical trials selected for inclusion used oral bLF as the main intervention, with co-interventions allowed. The trials needed to clearly state the daily dose given in both the intervention group and the control/comparison group. Trials that compared the intervention to a control or comparison group were eligible for inclusion, including those using oral iron preparations or any other form of conventional treatment for anemia. The change in mean Hb concentration values was considered the primary outcome measure. Randomized trials were the only type of study design that was eligible for inclusion.

The trials concerned participants with a mean Hb group value of ≤ 11.5 g/dL as a baseline characteristic. Only studies involving interventions on human subjects were considered, while those focused solely on the protective or potential effect of LF on healthy subjects were excluded. The publication year of the articles was not considered a restrictive factor in any case. Duplicate publications of the same trial were excluded. Only studies published in English were considered. Narrative reviews, dissertations or theses, conference papers, case studies, editorials, and letters were excluded.

### Study selection and data extraction

After the removal of irrelevant studies and duplicates, the full text of the retrieved articles was assessed to determine whether the content of the article and methodology pertained to the inclusion and exclusion criteria. Data was extracted independently by two authors (MC and AC) and tabulated using a table designed by the authors based on the ‘Cochrane Checklist of Items’ [12]. For the purpose of the systematic review, extracted information from the included articles referred to the authors and date of publication of the article, the participants’ characteristics that were included in the trial, their health condition, age, and mean baseline values for each group of participants included in the trial, the type of interventions used, the dose of the applied intervention in each group, the time frame of the intervention, and the main outcome of the trial shown as the mean value of Hb concentration for each group that completed the given intervention in the trial and its calculated p value. Disagreements were settled through mediation and discussion with a third author (KG).

### Quality assessment

The methodological quality of the included trials was assessed using the Jadad scoring scale. The Jadad scale is a validated tool for evaluating the methodological quality and risk of bias of randomized clinical trials [13]. It is based on the assessment of specific methodological qualities of clinical trials: randomization, blinding, and accountability of withdrawals and dropouts. Each trial may be scored within the range of 0–5 [14]. For the purposes of this meta-analysis, studies with a Jadad score of 3 or above were considered to have low methodological bias, while those with a Jadad score of 2 or less were considered to have high methodological bias.

### Data synthesis and statistical analysis

The inverse-variance weighted approach was used for continuous data meta-analysis with a Standardized Mean Difference (SMD) and 95% Confidence Interval (CI) to estimate the size of the effect calculated by pooling the SMD of each individual study based on the mean, standard deviations (SD), and sample size. SMD were chosen due to the variability in Hb concentrations between assays used by different laboratories and between measurements of Hb in different components of blood. Due to a lack of data, we only calculated a pooled effect size for the association between the lactoferrin group and ferrous sulfate on Hb concentration. We assessed heterogeneity among individual effect estimates and reported the P-value of the χ^2^-based Cochran Q test. The variation in estimates due to heterogeneity was quantified using the I^2^ metric for inconsistency and the I^2^ index (> 50% indicating significant heterogeneity) [15]. A random effects model was used to pool the results if I^2^ was greater than 50%; otherwise, a fixed-effect model was used [16]. To further explore sources of heterogeneity, we carried out subgroup analyses considering treatment duration. The presence of publication bias was examined by visual inspection of funnel plots and evaluated formally with Egger's regression asymmetry test [17, 18]. All statistical analyses were performed by STATA 14.0 software (STATA Corp. College Station, TX). A two-tailed *P* value < 0.05 was considered statistically significant.

## Results

### Study selection

The process of the search strategy and article selection for the systematic review and the meta-analysis is summarized in Fig. 1, following the PRISMA flow diagram 2020 [10]. A total of 158 articles were identified through database searches, with 25 from PubMed, 33 from CINAHL, 44 from Scopus, 13 from Cochrane and 43 from Embase. After the removal of duplicates, 111 article titles and abstracts were screened for relevance to the study’s field. Among these, 46 article titles were identified as potentially relevant. Among these, 25 article titles were excluded because only the abstract was published. The full manuscript of the remaining 21 article titles were assessed further for eligibility. Ultimately, only 10 articles met the selection criteria and were included in the systematic review. Additionally, 13 more articles were discovered by screening the citation lists of these included articles. Out of these, 8 met the inclusion criteria for this systematic review and were analysed.

One of the articles [19] included for review reported two separate trials—one involving non-pregnant women and the other involving pregnant women. We counted these as two separate trials. Therefore, a total of 19 randomized trials from 18 articles, yielded through database search and citation lists, were analyzed in this systematic review.

### Study characteristics

The 19 trials included in this systematic review were published between 2006 and 2022. Out of these, 5 of the trials reported were conducted in Italy [19–22] and 14 in Egypt [23–37]. The total number of participants who completed these trials was 2992 patients, with a mean Hb concentration level of ≤ 11.5 g/dL. Among them, 637 were children and adolescents, and 2355 were adults. There were 1353 participants in the LF intervention groups, and 1639 participants in the comparison/control groups. In terms of gender distribution, there were significantly more females, with 2516 (including 2266 women > 18 years and 250 children and adolescents < 18 years), as opposed to 326 males (including 89 men > 18 years and 237 children and adolescents < 18 years).

Among the 19 analyzed trials, the majority (53%—10 trials) focused on pregnant women with iron deficiency anemia (IDA) at or after 12 weeks of gestation, comprising a 2041 out of 2992 participants (939 in the intervention, 1102 in the control). This represented 68% of all participants involved in the trials [19, 21–23, 25, 26, 28, 29, 33–35]. Seven trials (37%) involved children aged over 2 [24, 27, 30–32, 36, 37], and one trial targeted cancer patients undergoing chemotherapy [20]. Additionally, four trials included 334 individuals with chronic health conditions (cancer, cerebral palsy, inflammatory bowel disease, obesity), all of whom had anemia (Hb < 11.5 g/dL) [20, 27, 32, 37].

Among the 1353 participants in the intervention groups, all received oral bLF. They were compared to the following groups: 1160 received ferrous sulfate [21, 22, 24–29, 33, 34, 38], 73 received ferric gluconate [20], 73 received ferrous fumarate [35], 30 received ferrous bisglycinate [30], 70 received ferric (III) hydroxide polymaltose [31, 37], 62 received iron polymaltose complex [30, 32], 33 received iron (III) hydroxide dextran complex [23], and 112 received a combination of LF and iron [24, 30, 31]. The bLF dosage ranged from 100 to 250 mg per day, and the treatment duration varied between 30 and 90 days. The study characteristics of the trials included in the systematic review are presented in Table 1.

**Table 1:** Characteristics of included trials

RT	Country	Sample	LF intervention group	Comparison group	Main findings
**Darwish et al.** (2018) [23]	Egypt	93 pregnant women, with IDA, > 18 years, gestational age between 14–28 weeks	60 participants LF oral sachets 200 mg per day	33 participants Τotal dose infusion of low molecular weight iron dextran (Iron (III)-hydroxide dextran complex) The dose for each participant was calculated according to the Ganzoni formula according to weight and iron stores	Both the iron and the LF therapy showed a rise in Hb concentration levels in participants. LF oral sachets can be used as an alternative to total dose infusion iron dextran supplementation
**El-Asheer et al.** (2021) [24]	Egypt	96 children, with IDA between 2–15 years old	32 participants 100 mg per day	32 participants Ferrous sulfate 6 mg/kg/day elemental iron administered orally once daily	There was a statistically significant difference in the increase of Hb concentration levels favouring LF treatment. Oral bovine LF was a more effective and safer alternative to oral ferrous sulfate. It has a better patient compliance and fewer side effects
**El-Hawy, Abd Al-Salam and Bahbah.** (2021) [30]	Egypt	120 children, with IDA, between 1–18 years old	30 participants 100 mg per day for children < 3 years old 200 mg per day for children > 3 years old	30 participants Iron bisglycinate chelate (FeBC) taken at a dose of 0.75 mg/kg/d 30 participants Iron polymaltose complex (IPC) (50mg/5 ml) at a dose of 6 mg/kg/d	The administration of LF with iron was more effective for increasing Hb concentration levels than the administration of iron alone
**El-Nasr et al.** (2021) [34]	Egypt	300 pregnant women, with IDA, second trimester (12 weeks of gestation onwards), with single fetus	150 participants combined intake of 200 mg of LF and 30 mg of iron per day	150 participants Dried ferrous sulfate 150 mg per day	Combined LF with ferrous sulfate was a more effective treatment, with a statistically significant difference, in increasing Hb concentration levels in pregnant women, than iron alone
**Kamal, Rezk and Hafez.** (2021) [31]	Egypt	150 children, with IDA, > 2 years of age	50 participants 200 mg per day	50 participants Ferric hydroxide polymaltose 6mg/kg/day 3X per day	There was a significant increase in Hb concentration levels in all three intervention groups. However, there was a significant difference favouring the LF and ferrous gluconate compound group. Therefore, LF with iron may be a more effective, alternative, treatment for increasing Hb concentration levels than the traditionally used iron alone
**Macciò et al.** (2010) [20]	Italy	148 cancer patients undergoing chemotherapy, ≥ 18 years old, histological diagnosis of a solid tumor at an advanced disease stage (stage III–IV)	75 participants s.c. rHuEPO-β, 30,000 UI once weekly and LF 200 mg/day	73 participants s.c. rHuEPO-β, 30,000 UI once weekly and ferric gluconate (125 mg i.v. weekly)	There was no significant difference in the mean Hb increase between the LF arm and the intravenous iron group. LF and i.v iron exhibited similar efficacy for increasing Hb concentration levels, in cancer patients undergoing chemotherapy
**Nappi et al.** (2009) [21]	Italy	97 pregnant women, with IDA, gestational age > 12 weeks and < 36 weeks, singleton pregnancy	49 participants 200 mg per day	48 participants Ferrous sulfate 100 mg of iron at the daily oral dose of one tablet of 520 m	Both the LF and the iron groups showed a significant increase in Hb concentration levels. However, there were no statistically significant differences between both treatments. Bovine LF had the same efficacy as ferrous sulfate
**Omar et al.** (2021) [32]	Egypt	66 children, with Cerebral Palsy and who met the WHO diagnostic criteria of IDA, aged between 1–10 years	34 participants oral bLF (30% iron-saturated) 200 mg per day	32 participants Iron Polymaltose complex at a dose of 6 mg/kg/day of elemental iron in 2 divided doses	Both the LF and the iron groups showed a statistically significant increase in Hb concentration levels. Bovine LF was effective and superior to the effect of iron hydroxide polymaltose complex, with fewer side effects, for children with cerebral palsy, suffering with IDA
**Paesano et al.** (2006) [22]	Italy	205 pregnant women, suffering from ID and IDA, in different stages of pregnancy	107 participants 200 mg per day	98 participants Ferrous sulfate 520 mg tablet dose containing 156 mg of elemental iron per day	Hb concentration levels showed a significant increase in all groups of women. However, in women receiving bovine LF treatment the mean values of Hb were higher than those in the ferrous sulfate group
**Paesano et al.** (2010) [19]	Italy	60 pregnant women, with mild IDA, third trimester of gestation	30 participants oral bLF (30% iron saturated) 200 mg per day	30 participants Ferrous sulfate 520 mg tablet dose containing 156 mg of elemental iron per day	Women treated with bovine LF showed a statistically significant increase in Hb concentration levels. Women treated with ferrous sulfate showed an increase in Hb concentration levels, but values did not reach statistical significance
**Paesano et al.** (2010) [19]	Italy	166 non-pregnant women of childbearing age, aged between 19–45 years old, with mild IDA	90 participants oral bLF (30% iron saturated) 200 mg per day	76 participants Ferrous sulfate 520 mg tablet dose containing 156 mg of elemental iron per day	Women treated with bovine LF showed a statistically significant increase in Hb concentration levels, in contrast to women treated with ferrous sulfate
**Rezk et al.** (2016) [33]	Egypt	200 pregnant women, with IDA, second trimester of gestation, single fetus	100 participants 250 mg per day	100 participants Dried ferrous sulfate capsules 150 mg per day	The total increase in Hb concentration levels in the LF group was higher than that compared to the ferrous sulfate group. Bovine LF was more effective than ferrous sulfate, with fewer gastrointestinal side effects and better patient compliance to treatment
**Hemeda et al.** (2018) [35]	Egypt	146 pregnant women, with IDA, gestational age > 14 weeks	73 participants 200 mg per day	73 participants Ferrous fumarate 350 mg iron (115 mg elemental iron) per day	The increase in Hb concentration levels was statistically significantly higher in the LF group in comparison to the iron fumarate group. Bovine LF was more efficacious than ferrous fumarate in the treatment of patients with a low Hb concentration profile. It is safer and has a better patient compliance to treatment
**El-Khawaga and Abdelmaksoud** (2019) [36]	Egypt	85 primary school children, with IDA, primary school age, between 6–12 years	43 participants 200 mg per day	42 participants Elemental iron 6 mg/kg/day	There was a statistically significant increase in Hb concentration levels in the LF group of patients compared to the elemental iron group. LF was a more effective and a safer alternative for treating patients with a low Hb concentration profile than elemental iron
**Atia et al.** (2021) [37]	Egypt	40 obese children and adolescents, with IDA, aged between 6–18 years	20 participants 100 mg per day	20 participants Ferric hydroxide polymaltose 6 mg/kg/day	There was significant elevation in the mean value of Hb concentration after therapy with bovine LF compared to the mean value after oral iron therapy. Bovine LF was superior to ferric hydroxide polymaltose for the treatment of Hb concentration levels, in obese children, with less gastrointestinal side effects
**Fawzy Mohamed, Soliman Taha and Farrag Ismaeil Farag.** (2020) [25]	Egypt	200 pregnant women, with IDA, gestational age 24–32 weeks, singleton pregnancy	100 participants LF plus folic acid 100 mg per day	100 participants Dried ferrous sulfate 150 mg per day	Hb concentration levels significantly increased in both the LF and iron groups, but the increase was statistically significantly higher in the LF group. Bovine LF was more efficacious in improving Hb concentration levels and better tolerated, with better patient compliance to treatment
**Bayoumy, Ragab and Elghareeb.** (2021) [26]	Egypt	140 pregnant women, with IDA, gestational age < 28 weeks, singleton pregnancy	70 participants 200 mg per day	70 participants Ferrous sulfate 200 mg per day	The increase of Hb concentration levels was significantly higher in the LF group compared to the ferrous sulfate group. Bovine LF was more effective than ferrous sulfate in improving Hb concentration levels and more tolerable
**Ali et al.** (2015) [29]	Egypt	600 pregnant women, Hb concentration level ≥ 10.5, gestational age 20–32 weeks, singleton pregnancy	200 participants 250 mg per day	200 participants Ferrous sulfate 150 mg per day 200 participants Amino acid chelated iron 15 mg per day	Hb concentration levels showed an increase in all groups after treatment. The difference in the mean Hb values among groups were statistically insignificant. LF was equally effective in improving Hb concentration levels as the other two trialled oral iron preparations
**El Amrousy et al.** (2022) [27]	Egypt	80 children with inflammatory bowel disease, suffering with IDA, aged between 5–18 years	40 participants 100 mg per day	40 participants Ferrous sulfate 6mg/kg/day	Hb concentration levels were significantly increased in both groups. However, the LF group had a significantly higher mean Hb value than the ferrous sulfate group. LF was an effective alternative to iron treatment with fewer side effects, in children with inflammatory bowel disease

*RT* Randomized trial, *LF* Lactoferrin, *bLF* Bovine lactoferrin, *IDA* Iron deficiency anemia, *Hb* Hemoglobin

### Methodological quality

According to the Jadad scoring scale, there was a variation in the methodological bias of the studies included in the meta-analysis, as detailed in Table 2. Studies with a Jaded score of 3 or above were considered to have low methodological bias, while those with a Jadad score of 2 or less were considered to have high methodological bias. Out of the included studies, five received 1/5 rating [29–31, 36, 37], six received a 2/5 rating [19, 20, 22, 25, 34, 35], six received a 3/5 rating [23, 24, 26–28, 32, 33], and 1 received a 4/5 rating [21]. Overall, 8 out of 18 studies were considered to have low methodological bias, while 10 out of 18 studies were considered to have high methodological bias. It is evident in the scoring process (Table 2.) that the problem originates from the lack of description of the randomisation process by most authors. The major issue, however, is the non-blinding of both patients and researchers/healthcare practitioners, which can give rise to treatment bias and may eventually influence the results. Only one study (Nappi et al*.*, 2009) out of 18 studies described the application of an appropriate blinding process during the trial.

**Table 2:** Jadad scoring scale of methodological risk of bias

	Author (date)	Was the study described as randomised?	Was the randomization scheme described and appropriate?	Was the study described as double-blind?	Was the method of blinding appropriate?	Was there a description of dropouts and withdrawals?	**Jadad score 0–5**
1	Darwish (2018) [23]	1	1	0	0	1	**3**
2	El-Asheer (2021) [24]	1	1	0	0	1	**3**
3	El-Hawy (2021) [30]	1	0	0	0	0	**1**
4	El-Nasr (2021) [34]	1	0	0	0	1	**2**
5	Kamal (2021) [31]	1	0	0	0	0	**1**
6	Macciò (2010) [20]	1	0	0	0	1	**2**
7	Nappi (2009) [21]	1	0	1	1	1	**4**
8	Omar (2021) [32]	1	1	0	0	1	**3**
9	Paesano (2006) [22]	1	0	0	0	1	**2**
10	Paesano (2010) [19]	1	0	0	0	1	**2**
11	Rezk (2016) [33]	1	1	0	0	1	**3**
12	Hemeda (2018) [35]	1	1	0	0	0	**2**
13	El-Khawaga (2019) [36]	1	0	0	0	0	**1**
14	Atia (2021) [37]	1	0	0	0	0	**1**
15	Fawzy Mohamed (2020) [25]	1	0	0	0	1	**2**
16	Bayoumy (2021) [26]	1	1	0	0	1	**3**
17	Ali (2015) [29]	1	0	0	0	0	**1**
18	El Amrousy (2022) [27]	1	1	0	0	1	**3**

### Meta-analysis results of Hb concentration outcomes

Out of the 19 included trials, seven were eligible for meta-analysis focusing on the effectiveness of lactoferrin versus iron supplementation in patients with a low Hb concentration profile. These trials included a total of 1397 participants: 698 supplemented with ferrous sulfate, and 699 with oral bLF. The participants were pregnant women with IDA, at a gestational age of at least 12 weeks but not more than 36 weeks.

**Fig. 2:**
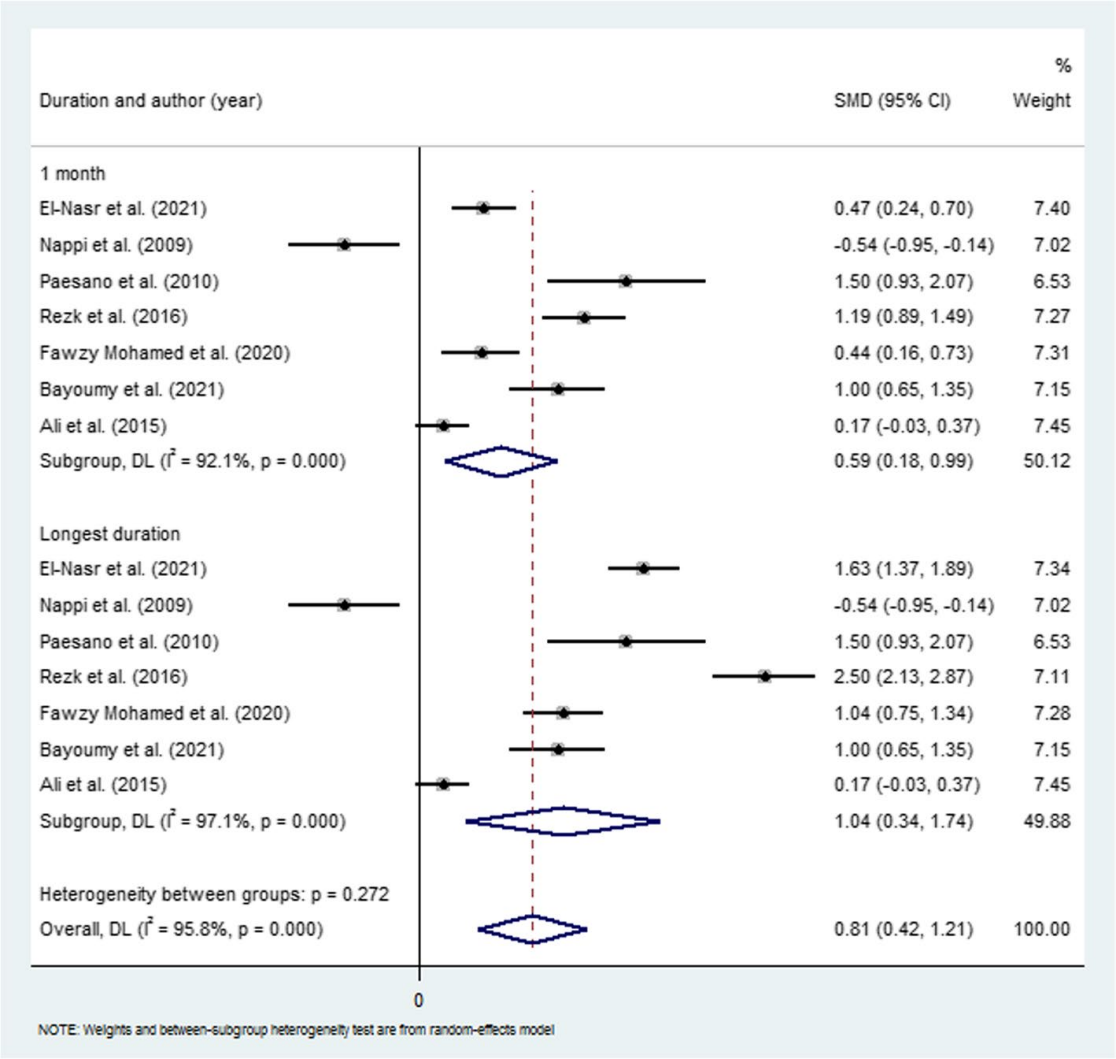
Forest plot of the standardized mean differences of the Hb concentration values

The overall pooled SMD, using a random-effects model, revealed a statistically significant increase in Hb concentration levels in the oral bLF group in comparison with the ferrous sulfate group (SMD 0.81, 95% CI: 0.42, 1.21, *p* < 0.0001, I^2^ = 95.8%, P heterogeneity < 0.001). Based on the random-effects model, the pooled SMD, after one month of treatment was 0.59 (95% CI: 0.18, 0.99, *p* = 0.004; I^2^ = 92.1%, P heterogeneity < 0.001). For those women who received the intervention for the longest duration, the pooled SMD, was 1.04 (95% CI: 0.34, 1.74, *p* = 0.004; I^2^ = 97.1%, P heterogeneity < 0.001). The results of the meta-analysis regarding the effectiveness of bLF compared to ferrous sulfate supplementation in pregnant women with low Hb concentration are presented in Fig. 2.

### Publication bias

**Fig. 3:**
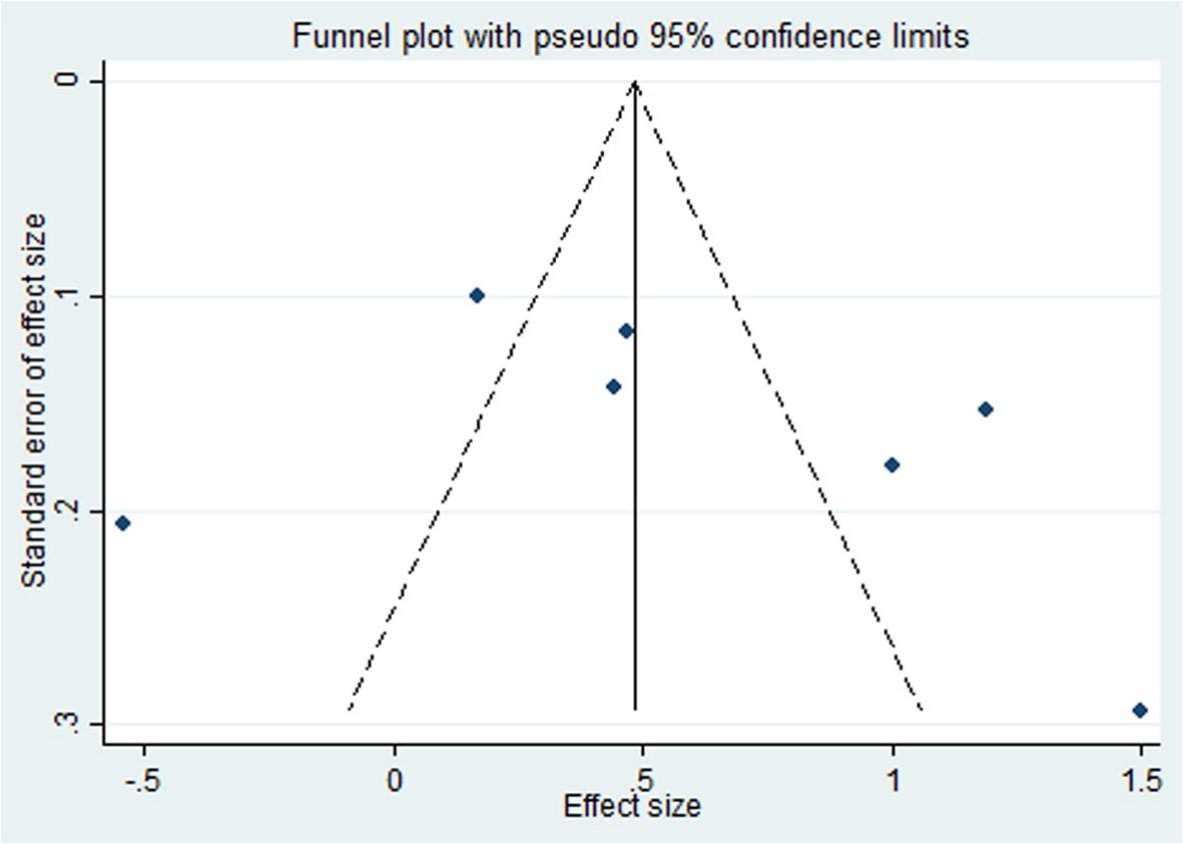
Funnel plot for publication bias evaluation (1 month treatment duration)

**Fig. 4:**
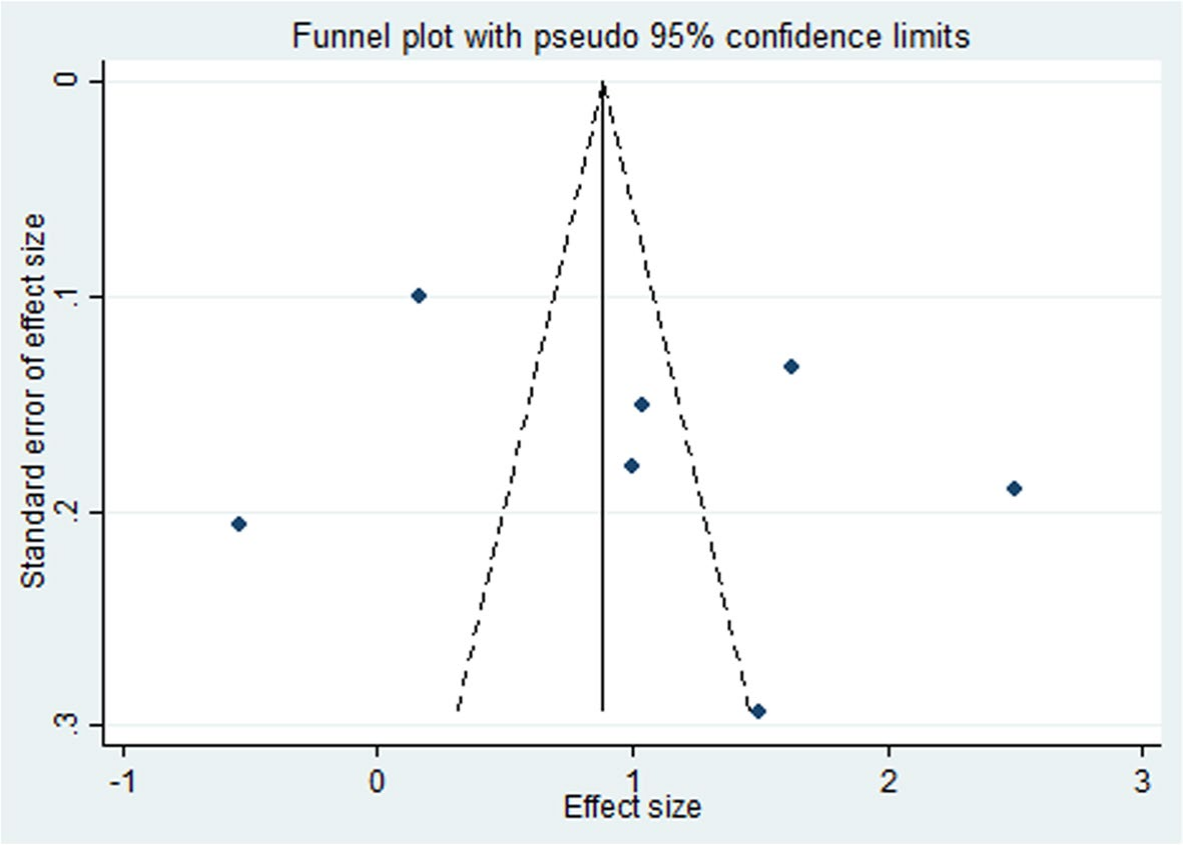
Funnel plot for publication bias evaluation (Longest treatment duration)

Funnel plots (Fig. 3 and Fig. 4) were used for the visual inspection of asymmetry or outliers for the purpose of determining publication bias. The shape of the funnel plots indicated data asymmetry. The Egger asymmetry test revealed no evidence of publication bias (P _Egger’s test_ P > 0.05).

## Discussion

The present systematic review and meta-analysis evaluated the effectiveness of oral bLF versus iron supplementation for patients with a low hemoglobin. It has been found that the levels of Hb concentration in different populations with varying health conditions undergo a moderate to significant change after treatment with all types of trialed interventions, including both iron and lactoferrin treatment, in both the intervention group and the comparison group. The trend of the results appears to favor bLF treatment. In all 19 randomized trials included in this study, bLF has been suggested as an effective and safe alternative to iron supplementation by the authors. Our meta-analysis demonstrated a statistically significant increase in Hb concentration favoring the oral bLF group over the ferrous sulfate group, with an overall pooled SMD of 0.81 (95% CI: 0.42, 1.21, *p* < 0.0001, I^2^ = 95.8%, P heterogeneity < 0.001). While several studies conducted, mainly on pregnant women [19, 22, 25, 26, 33, 35] and children [24, 27, 32, 36, 37], support this result, stating that LF showed a statistically significant increase in Hb concentration levels, compared to those in the iron group, some state that there were no statistically significant differences in the mean Hb increase between both treatments and that both treatments can be considered equally effective in improving Hb concentration levels [21, 29, 20].

For those who received the intervention for the longest duration, the pooled SMD was 1.04 (95% CI: 0.34, 1.74, *p* = 0.004; I^2^ = 97.1%, P heterogeneity < 0.001). Based on these results, subgroup analysis at the longest treatment duration suggests a slightly more significant effect compared to the one-month intervention, favoring bLF treatment. This finding warrants further investigation to determinate the optimal treatment duration. It is possible that daily bLF treatment for more than one month may lead to a more substantial increase in Hb concentration in the blood. This, in turn, may suggest that patients might need multiple cycles of LF treatment to significantly elevate their Hb concentration.

Several studies, included in this systematic review, propose that the Hb concentration enhancing effect of LF is attributable to its anti-inflammatory properties. More precisely, Paesano et *al.,* 2010 [38], investigated this hypothesis by measuring the fluctuations in pro-inflammatory and inflammatory markers, IL-6, CRP, ESR, in both the LF and iron populations recruited in their trials. It was observed that serum IL-6 levels were significantly decreased in gravidas with IDA at third trimester, who were treated with bLF, from a mean of 34.0 ± 8.0 pg/ml to 12.0 ± 10.0 pg/ml (*p* < 0.0001). The mean value of IL-6 increased in patients who were treated with ferrous sulfate, from a mean of 33.0 ± 13.0 pg/ml to 52.0 ± 13.0 pg/ml. This could possibly suggest that increased levels of the pro-inflammatory cytokine IL-6 in serum may be associated with lower levels of serum Hb mean value. This same effect was observed in two other included studies, involving children suffering from obesity and inflammatory bowel disease [27, 37]. This metanalysis analyzed the mean Hb values of pregnant women. Pregnancy is characterized by inflammatory processes which are normal and condition the course of physiological processes and body changes that take place during pregnancy till childbirth [39]. Therefore, it could be that the anti-inflammatory activity of LF plays a role in the modulation of Hb in pregnancy as demonstrated by Paesano et al*.* (2014) in anemic pregnant women affected from hereditary thrombophilia [40].

It is well established that inflammation exerts negative effects on iron homeostasis. Under inflammatory conditions ferroportin is downregulated thus impairing iron release into blood plasma. Hepcidin synthesis is controlled by IL-6, among other factors. Elevated levels of the pro-inflammatory cytokine IL-6, elevates hepcidin which in turn interacts with ferroportin at the cell surface, impairing iron release into plasma, thus causing hypoferremia [41].

Perhaps, a deeper inside into the mechanism of effect of LF can be concluded from previously published data taken from healthy individuals. In a randomized double blind study, Koikawa et al*.,* 2008 [42], recruited sixteen female long distance runners, with a healthy mean Hb value before treatment, divided into two groups (control and intervention). It was observed that there was no rise or significant difference in Hb mean values, (from 13.1 ± 0.8 g/dl to 13.0 ± 0.6 g/dl), after treatment with LF for 8 weeks, whilst the daily intake of oral iron supplementation was a constant in both groups. Furthermore, the rest of the hematology parameters examined (ferritin, serum iron, red blood cell count) didn’t show any significant change either. This evidence suggests that the therapeutic potentiality of LF with regard to Hb seems to be attributable to its capacity to modulate iron homeostasis. The failure of LF to increase blood parameters in healthy subjects is supporting evidence to this statement. Data demonstrate that lactoferrin may be a modulating agent in iron homeostasis by targeting the IL-6, Hepcidin, Ferroportin interplay [8].

A high patient compliance was recorded in participants receiving the LF treatment. Bayoumy, Ragab and Elghareeb, 2021 [26] and Hemeda et al*.,* 2018 [35], highlighted its statistical significance (*p* = 0.009 and *p* < 0.05 respectively). As compliance to treatment is always a prerequisite for optimum results, a higher adherence to LF treatment over iron supplementation may further explain the more favorable results of LF supplementation over oral iron supplementation. These results may be credited to the fact that fewer side effects to no side effects were reported with LF intervention than conventional iron supplementation, with significant difference (*p* < 0.001 and *p* < 0.05) [24, 33].

### Strengths and limitations

To our knowledge, this systematic review and meta-analysis represents the first evaluation of data from randomized clinical trials regarding the impact of LF on Hb concentrations in non-healthy patients, of both genders and different population groups. However, there are some limitations to this study. It only considered articles published in English, potentially excluding similar eligible studies in other languages. Although the study's overall quality of evidence suggests a potential risk of bias, it's important to consider that the study also had a high level of heterogeneity at 95.8%, which indicates a significant level of variability. This level of variability indicates inconsistent treatment results. This heterogeneity may stem from variations in treatment types, product formulation, timeframes, and population characteristics, as well as variations in the methods used by the researchers or even bias. Furthermore, all included studies were conducted in Italy and primarily in Egypt, limiting the demographic representation of the results. While the results of this systematic review and meta-analysis were not intended to support the introduction of a guideline, treatment protocol or treatment recommendation, nor to be used as a basis for accurate decision-making by healthcare providers, readers should be aware that these limitations may impact the reliability of results, and therefore, it is advisable to interpret these results with caution.

## Conclusion

This systematic review and meta-analysis provides representative data on the effectiveness of oral LF at doses of 100–250 mg/day, compared to conventional iron preparations in patients with low hemoglobin levels. The evidence suggests that bLF is an effective intervention for patients with low Hb concentration profiles, particularly in patients with inflammatory conditions. As a safer option and with high compliance evidence, lactoferrin can serve as an iron replacement treatment for patients who may be experiencing adverse side effects due to iron intake. Lactoferrin's ability to reduce pro-inflammatory cytokines seems crucial in regulating and restoring Hb levels. Reducing inflammation could potentially serve as the the first step toward normalizing Hb concentration levels in patients with low hemoglobin and inflammation-related conditions.

Future studies need to focus on reducing the risk of bias in their clinical trials to improve the methodological quality of the studies. This will help ensure the validity of the results and maintain the credibility and integrity of the study. Furthermore, it is important to examine whether product formulation, i.e. different types of lactoferrin preparations, affect Hb concentration differently. This will determine if different types of lactoferrin preparations are a confounding variable in such studies and may explain the high heterogeneity of results observed so far.

## Data Availability

The datasets used and/or analyzed during the current study are available from the corresponding author on reasonable request.

## Abbreviations

blf: Bovine Lactoferrin
Hb: Hemoglobin
CRP: C-reactive protein
ESR: Erythrocyte sedimentation rate
LF: Lactoferrin
IDA: Iron Deficiency anemia
IL-6: Interleukin 6
SMD: Standardized mean difference
SD: Standard deviation
CI: Confidence interval
